# Ultrasensitive Detection of Pathogenic *Bacteria* by Targeting High Copy Signature Genes

**DOI:** 10.3389/fvets.2022.889419

**Published:** 2022-04-29

**Authors:** Qiao Dong, Jingjing Chen, Qingqing Wei, Jinling Liu, Guoshun Shen, Baoshan Liu, Huan Zhang, Yuanzhi Wang, Zeliang Chen

**Affiliations:** ^1^Key Laboratory of Livestock Infectious Diseases, Ministry of Education, Shenyang Agricultural University, Shenyang, China; ^2^School of Medicine, Shihezi University, Shihezi, China; ^3^First Peoples Hospital of NingYang, Taian, China

**Keywords:** bacteria, IS711, high copy gene, signature gene, ultrasensitive detection of bacteria

## Abstract

Bacterial load in clinical samples is relatively low and difficult to detect. Improvements in assay sensitivity will greatly reduce false negative results and contribute to more accurate diagnoses. In the present study, we present a new strategy to improve the sensitivity of a nucleic acid assay by detecting the presence of a multi-copy gene. By using *Brucella* as a test model, we screened the genome and identified IS711 as a multiple copy gene. Distribution analysis of insertion sequence IS711 among different species and strains showed that each of the strains have 5 to 13 copies of IS711. Compared with the BMEI1001, BMEI0775 and BMEI0027, the assays of high copy genes IS711 showed higher sensitivity and is an ideal high copy signature gene for *Brucella*. Detection of clinical samples with assays targeting the signature genes showed that IS711 exist in higher concentrations than BMEI1001, BMEI0775 and BMEI0027. In addition, IS711 assay is more sensitive than other signature genes assay. Analysis of several other pathogenic bacteria successfully identified high copy number genes that could be used as signature genes. Therefore, this strategy of targeting high copy signature genes represents a universal strategy for the ultrasensitive detection of bacteria.

## Highlights

- In this study, *Brucella* was used as a test case to present a new strategy which proves sensitivity of nucleic acid assay by targeting multi copy gene.
- IS711 was identified as a multiple copy gene, and the assays targeting IS711 showed higher sensitivity for the same quantity of bacteria load compared with single copy target gene.
- Detection of clinical samples with assays showed that IS711 exist in higher concentrations than single copy target gene.

## Introduction

Bacterial pathogens cause numerous diseases that are refractory to diagnose and cure. Extensive application of antibiotics has led to the emergence of multi-drug and pan-drug resistant bacterial pathogens, making treatment more challenging. Rapid and sensitive diagnosis of these infectious diseases is of great importance for timely treatment and prevention of an outbreak (1). Molecular diagnostic assays, especially those targeting nucleic acids of pathogens, are the most widely used methods (2). However, for bacterial pathogens, particularly those that survive inside host cells and cause chronic infections, bacteria numbers in clinical samples are relatively low, presenting a significant challenge for detection. Although many highly sensitive methods have been developed to detect bacterial pathogens, their sensitivities are still restricted by low concentration of the pathogens in clinical samples (3, 4). Low sensitivity usually results in a false negative result, which may cause a delay in diagnosis or a misdiagnosis. Development of a new strategy that increases the detection sensitivity for bacteria pathogens will be of great value for accurate diagnosis of bacterial infectious diseases.

To improve the sensitivity of molecular detection of bacterial pathogens, we have designed a strategy that targets signature genes with highly abundant transcripts. With this strategy, it is possible to improve the sensitivity 10–100 fold (5). However, we have found that this strategy has two limitations: reverse transcription is required and RNA degradation in samples influences the sensitivity. RNA in bacteria is vulnerable to degradation in clinical samples (6). To make this strategy feasible for bacterial pathogen detection, the first step is to identify multi-copy signature genes. Previous studies have shown that *Brucella* is an intracellular bacterium that survives in host cells in low copy numbers (7, 8). Therefore, it is sometimes difficult to ensure high sensitivity.

Genomic DNA of bacterial pathogens is relatively stable in clinical samples, and the detection of bacterial DNA is not influenced by the factors that affect RNA detection. Building on our transcript detection strategy, we hypothesized that it might be possible to improve sensitivity by targeting DNA sequences that are in high abundance, and we selected the bacterial DNA for detection. In this study, we validate this hypothesis and demonstrate that this method can be a universal procedure for the sensitive detection of bacteria.

## Materials and Methods

### Identification Procedures of High Copy Signature Genes

Whole genome and individual gene sequences of *Brucella melitensis* strain 16M were downloaded from GenBank database (Accession No. AE008917 and AE008918) and saved in FASTA format. Gene sequences were searched against genome sequence by using the online BLASTN (two or more sequences) tool. The results were downloaded as CSV files and subsequently analyzed using Microsoft Excel. Genes that aligned to genome sequence more than once with identities over 80% were considered multi-copy gene candidates. Candidate genes were searched with BLAST against nucleic acid databases to test their uniqueness for the *Brucella* genus.

### Analysis of IS711 Sequences

The sequence of IS711 was compared against genome sequences of different *Brucella* species by using the online tool BLASTN. IS711 loci with identical adjacent genes were considered to be the same locus. BLAST results were downloaded as CSV files and then analyzed in Microsoft Excel. Insertion locations and numbers for each chromosome of the genomes were recorded and compared. Statistical analysis was performed with SPSS version 19. *P*-values <0.05 were defined as statistically significant.

### Bacteria Culture and Template Preparation

*Brucella melitensis* 16M was grown in tryptic soy broth (TSB) medium at 37°C to an OD_600_ of 1.0. For template preparation, 1 ml of bacterial culture was centrifuged at 12,000 × g for 5 mins, re-suspended in 200 μl ddH_2_O, and heat-denatured at 100°C for 10 min. Heat denatured suspension was centrifuged at 10,000 × g for 2 min and the supernatants were collected and stored at −20°C for future use. To calculate the bacteria concentration, 1 ml of bacterial culture was centrifuged at 4,000 × g for 5 min and then re-suspended in 200 μl TSB, which was then serially diluted 10-fold and plated on tryptic soy agar (TSA) plates. Bacteria were counted after 3 days of cultivation at 37°C.

### Quantitative PCR

The plasmid or sample template was subjected to quantitative PCR (qPCR) using the 2 × SYBR I mix (Takara, China) with a 20 μl volume composed of 10 μl 2 × SYBR I, 0.6 μl of each primer, and 6.8 μl of ddH2O. The qPCR reaction was performed on an IQ5 real-time PCR detection system (Bio-Rad). The reaction condition was as follows: 5 min at 95°C, followed by 40 cycles of 15 s at 95°C, 30 s at 60°C. Specificity was evaluated by detection of other bacterial samples, including *Proteus mirabilis, Streptococcus pneumonia, Escherichia coli UPEC, Klebsiella pneumoniae, and Staphylococcus aureus*.

### Detection Efficiency Comparison

To validate whether the high copy target genes could generate higher detection sensitivity, we selected four genes with varying copy numbers for further analysis. A high copy recombinase gene BMEI1001 (seven copies), low copy gene BMEI0775 (two copies), insertion sequence IS711 (six copies), and single copy gene BMEI0027 (one copy) were selected for comparison (**Figure 2A**). Primers for these genes were designed and synthesized (Beijing Sangon Biotech Company Ltd., Beijing, China; sequences shown in Supplementary Table 1). The target fragments were PCR amplified with primers from the templates for the 16M bacterial strain, and TA cloning was used to insert the sequence into a pMD-18T vector (Takara, China) to generate recombinant plasmids, which were then used as templates for assay development and sensitivity evaluation.

### Ethic Statement and Clinical Sample Detection

Suspect brucellosis patients were enrolled at Brucellosis Hospital of Plague and Brucellosis Prevention and Control Base. Blood samples were collected in sodium citrate and stored at −20°C during routine laboratory diagnosis at affiliated brucellosis hospital of Plague and Brucellosis Prevention and Control Base (Baicheng, China). Brucellosis patients were diagnosed based on serological assay standard agglutination test (SAT), clinical symptom and epidemic information. Cut-off value for SAT assay was 1:100. We collected 20 blood samples from patients with serologically confirmed brucellosis. This study was carried out in accordance with the recommendations of Ethics Committee of Plague and Brucellosis Prevention and Control Base with written informed consent from all subjects. All subjects gave written informed consent in accordance with the Declaration of Helsinki. Templates were prepared essentially as follows: 0.5 ml of blood was resuspended in 1 ml of erythrocyte lysis solution (320 mM saccharose, 5 mM MgCl_2_, 1% Triton X-100, 10 mM Tris HCl [pH 7.5]), mixed, and centrifuged at 15,000 g for 2 min. The supernatant was discarded, and the pellet was washed with 1 ml of PBS. The pellet was re-suspended in water and lysed at 100°C for 10 mins. The lysis solution was centrifuged 10,000 g for 5 min and supernatant was used as template. qPCR detection was performed as described above. The experimental protocols were approved by Ethics Committee of Institute of Disease Control and Prevention, Academy of Military Medical Sciences.

## Results and Discussion

**Figure 1:**
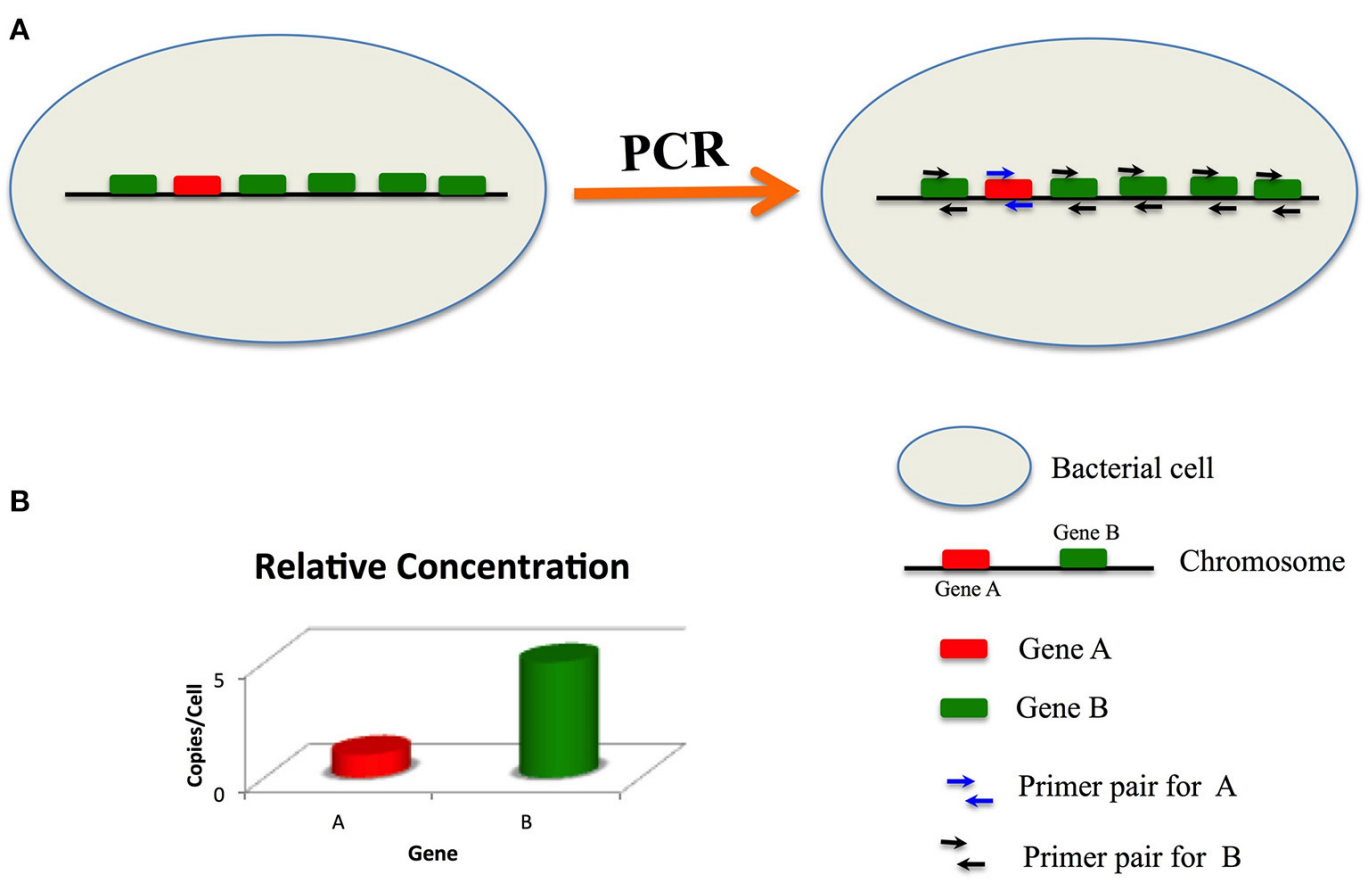
Principle of sensitive detection by targeting multi-copy signature genes. In a bacteria cell, there is one copy of gene **(A)** and five copies of gene **(B)** primers for gene **(A)** have only one binding site, while primers for gene B have five binding sites. If the amplification efficiencies of **(A)** and **(B)** are identical, then the sensitivity of detection of gene **(B)** is five times that of gene **(A)**.

We hypothesized that high copy target signature genes would improve the sensitivity of bacterial detection. Based on our hypothesis, we designed a new strategy to improve the sensitivity of nucleic acid detection. As shown in Figure 1, there is one copy of gene A and five copies of gene B in a bacterial genome. If the PCR amplification efficiencies for gene A and gene B are identical, then the sensitivity of the PCR assay targeting gene B will be five times of that greater than that targeting gene A.

We used *Brucella* as a test model because its numbers in clinical samples are relatively low and detection of this pathogen is difficult. High copy-number genes of *B. melitensis* strain 16M were identified through alignment of individual genes to its genome sequence. Genes with two or more alignment sites in the genomes are listed in Supplementary Table 2. Based on the alignment results, multi-copy genes were identified. As shown in Table 1, a total of 38 genes were found to have multiple copies: 13 genes (34.2%) have seven copies, five genes (13.2%) have three copies, and 20 genes (52.6%) have two copies. Interestingly, 24 (63.16%) of these high copy genes encode transposases. All of the seven copy genes and three (out of four) of the three copy genes are transposase genes. This type of high copy gene exists extensively in various bacteria. Sequences of these high copy genes were searched with BLAST against nucleotide sequences in the Genbank database. All of these sequences are conserved among different species of *Brucella* but show very low similarity with other sequences (data not shown), indicating that these high copy genes are specific for *Brucella* and could be used as signature sequences.

**Table 1:** Multi-copy genes in genome of *B. melitensis* 16M.

**Gene name**	**Gene length**	**Aligned length**	**Copy number**	**Genomic location** ^a^				**Description**
				**Chromosome**	**Strand**	**Start**	**End**	
BMEI1001	393	393	7	I	–	1043436	1043044	Transposase
BMEI1002	369	369	7	I	–	1043801	1043433	Transposase
BMEI1052	279	279	7	I	–	1093082	1092804	Transposase
BMEI1053	369	369	7	I	–	1093447	1093079	Transposase
BMEI1163	279	279	7	I	–	1210016	1209738	Transposase
BMEI1164	369	369	7	I	–	1210381	1210013	Transposase
BMEI1406	279	279	7	I	–	1458552	1458274	Transposase
BMEI1407	369	369	7	I	–	1458917	1458549	Transposase
BMEI1814	393	393	7	I	–	1852504	1852112	Transposase
BMEI1815	369	369	7	I	–	1852869	1852501	Transposase
BMEII0445	267	267	7	II	–	465790	465524	Transposase
BMEII0718	369	369	7	II	+	757373	757741	Transposase
BMEII0719	279	279	7	II	+	757738	758016	Transposase
BMEI1400	276	276	3	II	–	1454998	1454723	Transposase
BMEI1405	240	240	3	II	–	1458158	1457919	Transposase
BMEII0710	366	366	3	II	+	751312	751677	Transposase
BMEII0716	162	162	3	II	+	755120	755281	Transposase
BMEII0314	903	136	3	II	–	329914	329012	Anthranilate synthase
BMEI0168	2394	2394	2	I	+	162767	165160	Cell division protein ftsk
BMEI0742	1,176	1,221	2	I	+	768090	769265	Elongation factor tu (refseq)
BMEI0755	1,176	1,176	2	I	+	786042	787217	Protein translation elongation factor tu
BMEI0890	1,134	1,134	2	I	+	921879	923012	Queuine trna-ribosyltransferase
BMEI0902	231	231	2	I	+	935275	935505	Recombinase
BMEI0903	1,344	1,344	2	I	+	935903	937246	Hypothetical protein
BMEI0904	246	246	2	I	–	937936	937691	Hypothetical protein
BMEI0905	222	222	2	I	+	938429	938650	Hypothetical protein
BMEI0906	318	318	2	I	–	939309	938992	Hypothetical protein
BMEI1003	609	609	2	I	–	1044479	1043871	Queuine trna-ribosyltransferase
BMEI1399	309	309	2	I	–	1454879	1454571	Transposase
BMEI1411	309	309	2	I	–	1460411	1460103	Transposase
BMEI1412	276	276	2	I	–	1460530	1460255	Transposase
BMEI1659	246	246	2	I	+	1710657	1710902	Hypothetical protein
BMEI1660	1,344	1,344	2	I	–	1712690	1711347	Hypothetical protein
BMEI1873	1,518	367	2	I	–	1924407	1922890	Cell surface protein
BMEII0183	903	903	2	II	–	196444	195542	Transposase
BMEII0184	282	282	2	II	–	196722	196441	Transposase
BMEII0227	282	282	2	II	+	242480	242761	Transposase
BMEII0228	930	930	2	II	+	242758	243687	Transposase

a *Locations according to genomic annotation*.

The prevalence of high copy transposase genes implies that gene transposition might be common in *Brucella*. Mobilization has been observed in *Brucella*. Insertion sequences are the simplest mobilization elements in bacteria. In *Brucella*, one important insertion sequence, IS711 (also termed IS6501), has been identified (9, 10). IS711 has been shown to exist in *Brucella* genomes in multiple copies. The IS711 insertion location has been shown to be specific for *Brucella* species and has been used as a signature gene for *Brucella* identification. IS711 is also used for differentiating between the four main *Brucella* species (11, 12). IS711 has been used before in real-time PCR for *Brucella* detection (13). Assays of IS711 have been shown to be more sensitive than using omp31. However, the insertion locations and copy numbers of IS711 in different species of *Brucella* have not been systematically analyzed, and the idea of using this high copy gene based strategy has never been put forward.

**Figure 2:**
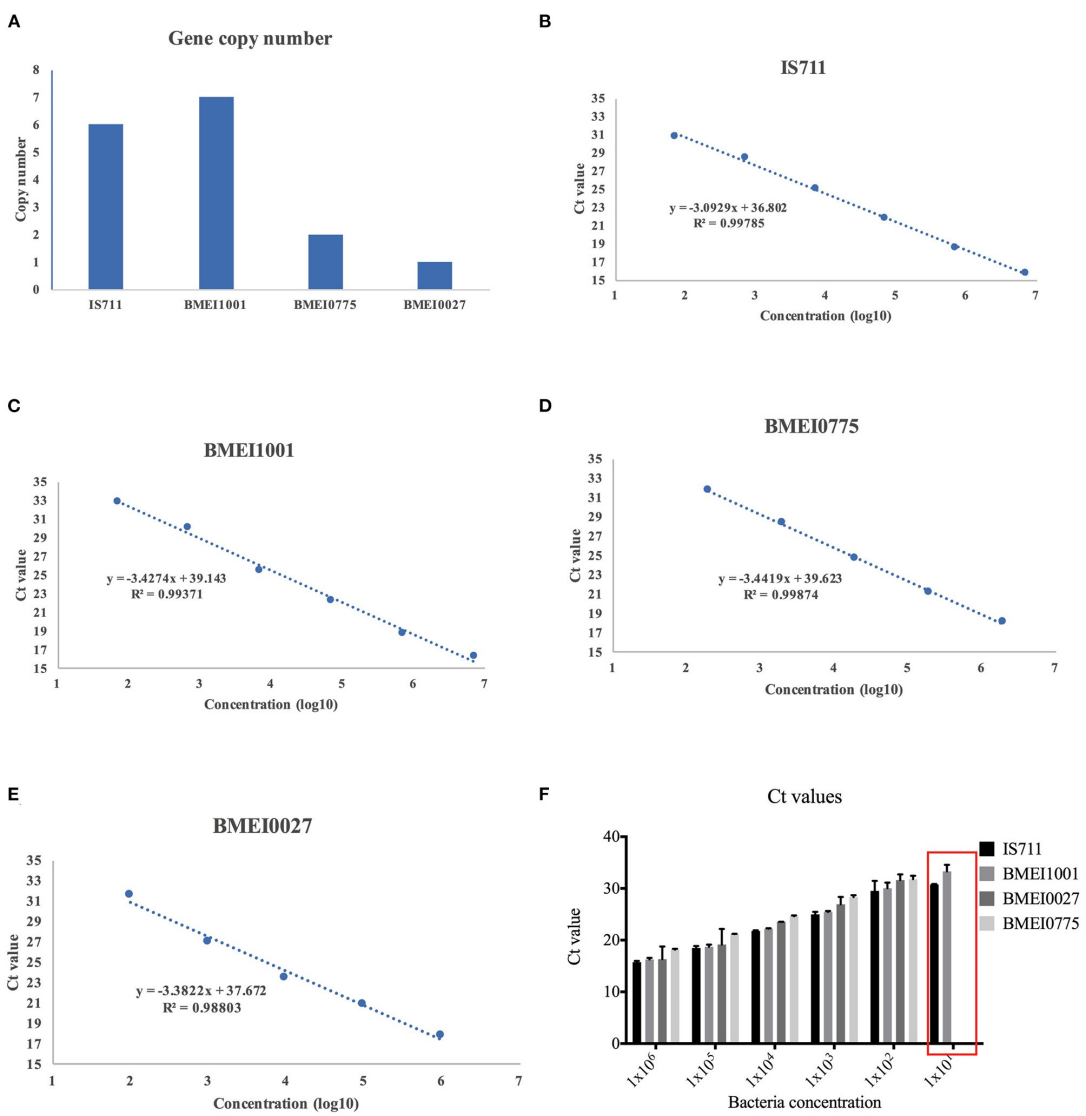
Detection efficiency of signature genes with varying copy number. Four candidate signature genes, IS711, BMEI1001, BMEI0775, and BMEI0027, with varying copy numbers were selected **(A)** and serial dilutions of bacteria culture were assayed with primers for the four genes. Quantification curves of IS711 **(B)**, BMEI1001 **(C)**, BMEI0775 **(D)**, and BMEI0027 **(E)** were generated. High copy genes IS711 and BMEI1001 showed higher sensitivity than low copy genes BMEI0775 and BMEI0027 **(F)**.

A recombinase gene BMEI1001 (seven copies), IS711 (six copies), BMEI0775 (two copies), and BMEI0027 (one copy) were selected (Figure 2A). Amplification of serial dilutions of 16M templates showed that the four genes have varying amplification windows. R^2^ values of the four assays were >0.99, implying that they have similar high amplification efficiencies. As shown in Figure 2, while high copy genes, BMEI1001 and IS711, could be amplified across six orders of magnitude dilutions of the template, low copy genes BMEI0775 and BMEI0027 could be amplified across only five orders of magnitude dilutions of the template (Figures 2B–E). Assays of high copy genes could detect the lowest concentration of bacteria tested, while assays of the low copy genes could not, indicating that detection of high copy genes have higher sensitivity (Figure 2F). Although both BMEI1001 and IS711 could be detected across six orders of magnitude dilution of the template, they differed from each other with respect to their C_t_ values. From the amplification curve, IS711 showed better amplification efficiency. For the lowest concentration, the C_t_ value of the IS711 assay was 30.7, which was lower than the C_t_ value of BMEI1001 (32.8). Interestingly, this is contrary to what was expected for the sensitivity assay. From these analyses, it can be concluded that IS711 is an ideal high copy signature gene for *Brucella*.

**Figure 3:**
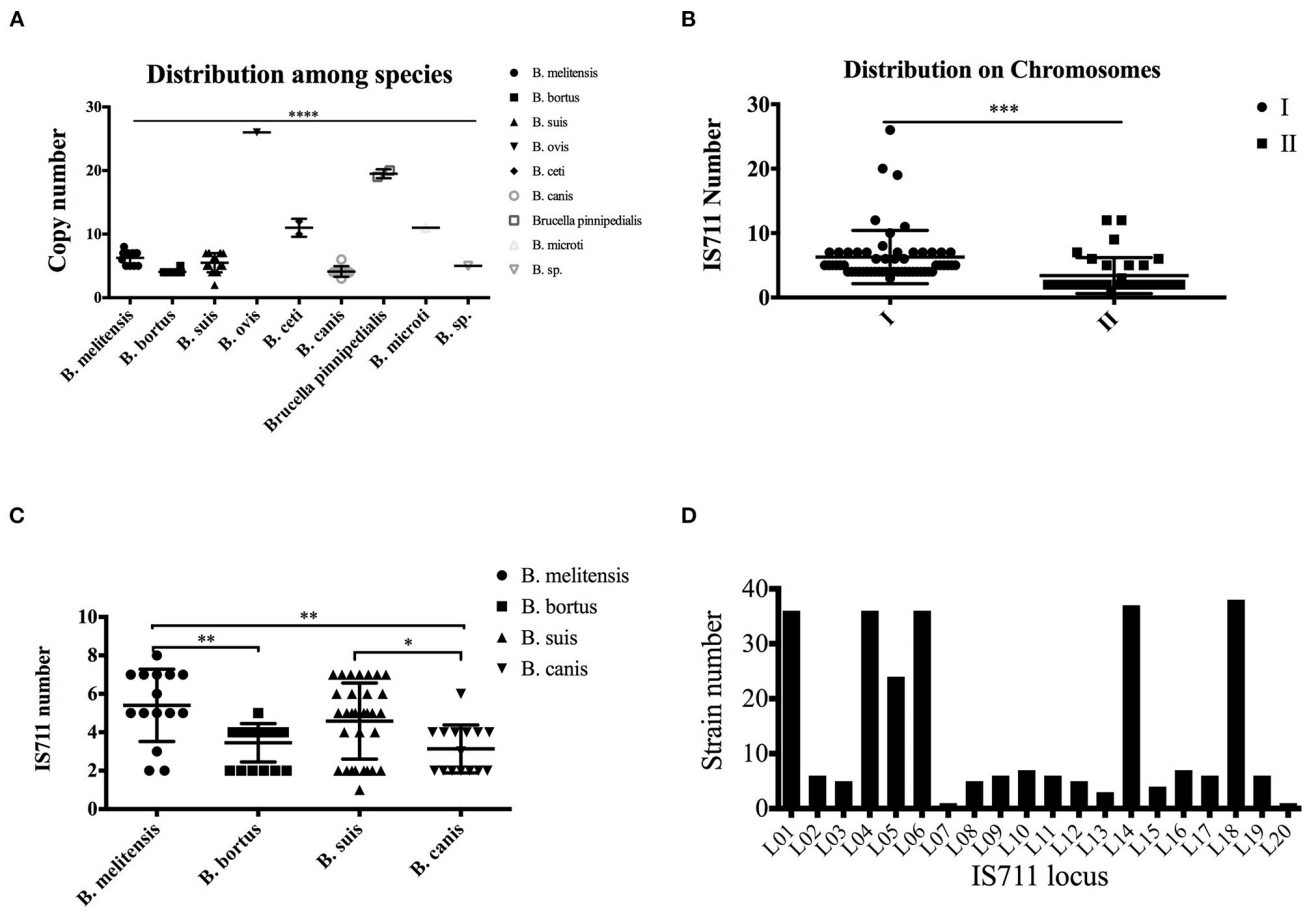
Differential distribution of IS711 among different *Brucella* species. Copy number of IS711 was calculated for each of the nine *Brucella* species. IS711 copy number varies significantly among the species **(A)**. Copy number of IS711 on chromosome I is significantly higher than that on chromosome II **(B)**. IS711 copy numbers were compared between the four species that infect humans. *B. melitensis* has a higher copy number of IS711 than *B. abortus* and *B. canis* **(C)**. A total of 20 IS711 locus were identified, and they are differentially distributed among the strains **(D)**. **** *P* < 0.0001, *** *P* < 0.001, ** *P* < 0.01, * *P* < 0.05.

We then analyzed the copy distribution of IS711 among *Brucella* species by examining whole genome sequences. Different *Brucella* species have different IS711 copy numbers in their genomes (Table 2 and Figure 3). In addition, different *Brucella* species have different BMEI1001, BMI0775 and BMEI0027 copy numbers in their genomes (Supplementary Tables 3–5). We found that BMEI1001 exists in multiple copies in various *Brucella* species. Moreover, the copy number of BMEI1001 is similar to IS711, and especially high copies in *B. melitensis* and *B. abortus* that are highly pathogenic to human and animals. Therefore, BMEI1001 may be a candidate high-copy signature target gene to replace IS711. Copy number of IS711 among *Brucella* strains ranged from two to 26, with a mean of 6.25 (95% CI 5.12–7.37). *B. ovis* has the highest copy number with 26, followed by 19.5 in *B. pinnipedialis*, 11.0 in *B. ceti* and *B. microti*, and 6.27 in *B. melitensis* (Figure 3A). *Brucella* strains have two chromosomes, so we analyzed the distribution of IS711 between the two chromosomes. Chromosome I is considered to be a conventional chromosome, while chromosome II is a large plasmid derived by unknown mechanisms. Generally, chromosome I has more copies of IS711 than chromosome II (Figure 3B). For *B. ceti* and *B. microti*, IS711 was found only on chromosome I. Although no copies of IS711 were found in chromosome II, the number of copies of IS711 in chromosome I was relatively high. Both *B. ceti* and *B. microti* have 11 copies of IS711 in chromosome I, which is higher than that of *B. abortus* (4.07), *B. melitensis* (6.27), and *B. suis* (5.5). Interestingly, IS711 copy number among species of *B. ovis, B. ceti, B. pinnipedialis*, and *B. microti*, which are not pathogenic for humans, is higher than that of *B. melitensis, B. abortus, B. canis*, and *B. suis*, which are pathogenic for humans (Figure 3A). Correlation of insertion sequence frequency with virulence variation has been observed in bacteria. At present, it is unclear whether the IS711 copy number is related to evolution of *Brucella* virulence, but there is a negative correlation of IS711 copy number and virulence for humans.

**Table 2:** Copy distribution of IS711 among different species.

**Species**	**Genome**		**Chromosome I**		**Chromosome II**	
	**Strain number**	**IS711 mean**	**Strain number**	**IS711 mean**	**Strain number**	**IS711 mean**
*B. melitensis*	11	6.27	11	6.17	6	2.33
*B. abortus*	14	4.07	14	4.07	6	2.00
*B. suis*	16	5.50	16	5.69	15	3.40
*B. ovis*	1	26.00	1	26.00	1	12.00
*B. ceti*	2	11.00	2	11.00	–	–
*B. canis*	8	4.12	8	4.13	7	2.00
*B. pinnipedialis*	2	19.50	2	9.50	2	10.50
*B. microti*	1	11.00	1	11.00	–	–
*B. spp*	1	5.00	1	5.00	1	2.00

IS711 has been shown to be inserted differentially among the four human pathogenic *Brucella* species, which is used as a signature for species identification (12). The PCR assay targeting IS711 location is termed as AMOS-PCR (used for *B. abortus, B. melitensis, B. ovis*, and *B. suis*). As expected, IS711 copy number was differentially distributed among the four species (Figure 3C). To further confirm the distribution of IS711 among different *Brucella* species, we analyzed its genomic location distribution. A total of 20 loci were identified among the tested strains. These loci have completely identical IS711 sequences, but have different adjacent genes. The 20 loci are distributed differently among these *Brucella* strains (Supplementary Table 6). While some of these loci exist in most of the strains, others exist in only a small number of the strains (Figure 3D). We further characterized these IS711 genes among the four species (Table 3). Locus 18 exists in all strains, while loci 20 and 7 were observed in only one strain. Based on the IS711 loci distribution, it is possible to screen potential loci that differ among different species. For example, locus 5 were deleted in *B. abortus*, loci 9–12 were observed only in *B. suis*, and locus 8 was only detected in *B. melitensis*. These differential distributions provide new potential signature candidates for species identification and differentiation.

**Table 3:** IS711 genomic location distribution among different brucella strains.

**Species**	**Strain**	**IS711 Locus**																				**Locus number**	**Total Copy**
		**1**	**2**	**3**	**4**	**5**	**6**	**7**	**8**	**9**	**10**	**11**	**12**	**13**	**14**	**15**	**16**	**17**	**18**	**19**	**20**		
*B. abortus*	63 75(NZ_CP007663.1)	1			1		1								1				1			5	5
	BDW(NZ_CP007681.1)	1			1		1								1		1		1			6	7
	BER(NZ_CP007682.1)	1			1		1								1				1			5	5
	BFY(NZ_CP007738.1)	1					1								1				1			5	5
	3196	1			1		1								1				1			5	5
	A13334	1			1		1								1		1		1			6	6
	9–941	1			1		1								1		1		1			6	6
	S19	1			1		1								1		1		1			6	6
	870	1			1		1								1				1			5	5
	C68	1			1		1								1				1			5	5
	86/8/59	1			1		1								1		1		1			6	6
	biovar Abortus 2308	1			1		1								1		1		1		1	7	7
	NCTC10505	1			1		1								1				1			5	5
	Human/AR/US/1981	1			1	1	1								1	1			1			7	7
*B. suis*	513UK	1			1	1	1			1					1				1			6	13
	BSP	1			1	1	1								1				1			6	6
	VBI22	1			1	1	1								1	1			1			7	8
	S2	1			1	1	1								1	1			1			7	7
	1330	1			1	1	1								1	1			1			7	7
	Bs364CITA				1	1	1				1	1	1	1	1			1	1	1		11	13
	PT09172	1			1	1	1			1	1	1	1		1			1	1	1		12	12
	PT09143	1			1	1	1			1	1	1	1		1			1	1	1		12	12
	Bs396CITA	1			1	1	1			1	1	1	1	1	1			1	1	1		13	13
	686	1			1	1					1				1				1			6	6
	ATCC 23445	1			1	1	1			1	1	1	1	1	1			1	1	1		12	13
	Bs143CITA	1			1	1	1			1	1	1			1			1	1	1		10	12
*B. melistensis*	16M	1	1		1	1	1								1				1			7	7
	NI			1	1	1	1		1						1				1			7	9
	M28	1	1	1	1	1	1		1						1				1			9	9
	M5-90	1	1	1	1	1	1		1						1				1			9	9
	ATCC 23457	1	1	1	1	1	1		1						1				1			9	9
	Ether	1	1		1		1	1											1			6	8
	63/9	1	1	1	1	1	1		1						1				1			9	10
*B. canis*	SVA13	1			1	1									1				1			6	6
	RM6/66	1			1	1	1								1				1			6	6
	ATCC 23365	1			1	1	1								1				1			6	6
	HSK A52141	1			1	1	1								1				1			6	6
	Oliveri	1				1	1								1				1			5	6

**Figure 4:**
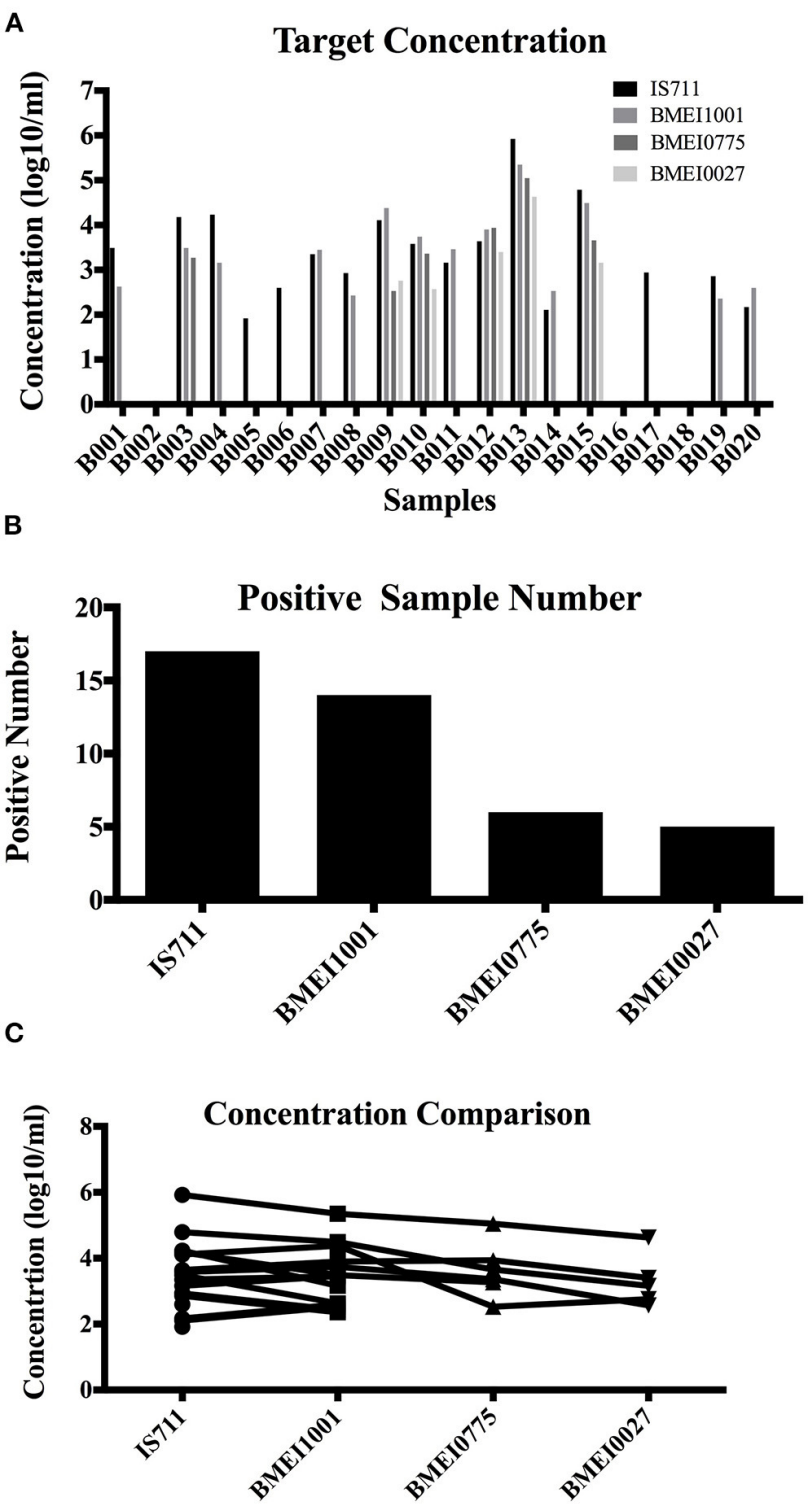
Detection of clinical blood samples of human brucellosis patients. A total of 20 samples from human brucellosis patients were tested with IS711, BMEI1001, BMEI0775, and BMEI0027 assays, and the target concentration was calculated for each sample. Target concentration **(A)** and positive number **(B)** of IS711 and BMEI1001 was higher than that of BMEI0775 and BMEI0027 **(C)**.

The improved detection performance by targeting high copy signature genes was tested in clinical sample detection. Samples were detected with the four assays and target concentration was calculated (Table 4 and Figure 4A). From the 20 samples, IS711 was detected in 17, followed by BMEI1001 in 14, BMEI0775 in six, and BMEI0027 in five (Table 4 and Figure 4B). For the four-assay positive samples, target concentrations (log^10^/ml) of IS711 and BMEI1001 were consistently higher than BMEI0775 and BMEI0027 (Table 4 and Figure 4C). Concentration of IS711 ranged from 1.92 to 5.92 (95% CI 2.88–3.94), for BMEI1001, it ranged from 2.58 to 5.35 (95% CI 2.913–.94). The concentrations of IS711 and BMEI1001 were similar. This further provides the possibility that BMEI1001 may become a candidate to replace high-copy target gene of IS711. Sequencing of the PCR products confirmed that the assays detected the correct target sequence (data not shown). These data indicate that high concentrations of high copy genes exist in clinical samples and assays to detect these genes are more sensitive and accurate.

**Table 4:** Detection results of 20 clinical blood samples from brucellosis patients.

**SampleID**	**Target concentration (log**_10_ **/ml)** ^a^				**SAT titer**	**Sequence confirmation**
	**IS711**	**BMEI1001**	**BMEI0775**	**BMEI0027**		
B001	3.49	2.63	ND	ND	1/200	Yes
B002	ND	ND	ND	ND	1/200	–
B003	4.18	3.49	3.27	ND	1/200	Yes
B004	4.23	3.16	ND	ND	1/200	Yes
B005	1.92	ND	ND	ND	1/200	Yes
B006	2.60	ND	ND	ND	1/200	Yes
B007	3.35	3.45	ND	ND	1/200	Yes
B008	2.93	2.43	ND	ND	1/200	Yes
B009	4.11	4.38	2.53	2.76	1/200	Yes
B010	3.58	3.74	3.36	2.57	1/200	Yes
B011	3.16	3.46	ND	ND	1/200	Yes
B012	3.64	3.90	3.94	3.40	1/200	Yes
B013	5.92	5.35	5.05	4.63	1/200	Yes
B014	2.11	2.53	ND	ND	1/200	Yes
B015	4.79	4.49	3.66	3.16	1/200	Yes
B016	ND	ND	ND	ND	1/200	–
B017	2.94	ND	ND	ND	1/200	Yes
B018	ND	ND	ND	ND	1/200	–
B019	2.86	2.36	ND	ND	1/200	Yes
B020	2.17	2.60	ND	ND	1/200	Yes
Control	ND	ND	ND	ND	–	–

a *Concentration was calculated with standard curve of each assay*.

*ND, not detected; –, not applicable*.

To further confirm whether this high copy signature gene strategy is feasible for other bacteria, we searched for high copy genes in other bacterial genera. Genome sequences of *Coxiella burnetii, Mycobacterium tuberculosis*, and *Mycobacterium bovis* were screened for high copy genes. As expected, high copy genes were successfully identified among the three species. Selected high copy genes are listed in Table 5. The copy number of these genes varied greatly. For *Coxiella burnetii*, copy number of these genes is as high as 20. For *Mycobacterium tuberculosis*, the highest copy number gene is IS6110 transposase, whose copy number is 16. For *Mycobacterium bovis*, the highest copy gene is IS1081 transposase. As was the case in *Brucella*, most of these high copy genes are insertion sequence related transposases. However, many high copy genes are hypothetical genes with unknown functions. We analyzed the sequence homologies of these high copy genes and found that all of them are unique to and conserved among the species. IS6110 has been used as a signature sequence for *Mycobacterium tuberculosis* diagnosis (14, 15). We chose two Mycobacteria because they are closely related. As expected, their high copy genes varied significantly. This further confirmed that insertion sequences are good signature candidates for bacteria detection and differentiation. Therefore, this high copy signature gene detection strategy is universal and could be extended to other bacteria.

**Table 5:** Selected multiple copy genes in genomes of *Coxiella burnetii, Mycobacterium tuberculosis* and *Mycobacterium bovis*.

**Species and strain**	**Gene locus**	**Copy number**	**Function description**
*Coxiella burnetii* RSA 493	CBU_1076	20	IS1111A transposase
	CBU_1090	20	IS1111A transposase
	CBU_1186	20	IS1111A transposase
	CBU_1269a	18	hypothetical protein
	CBU_1758b	16	hypothetical protein
	CBU_1716b	6	hypothetical protein
	CBU_1061a	5	hypothetical protein
	CBU_1555	3	ISAs1 family transposase
*Mycobacterium tuberculosis* H37Rv	Rv1756c	16	Transposase
	Rv1757c	16	Transposase for insertion sequence element IS6110
	Rv1763	16	Transposase for insertion sequence element IS6110
	Rv1764	16	Putative transposase
	Rv3343c	7	PPE family protein PPE54
	Rv2512c	6	Transposase for insertion sequence element IS1081
	Rv2666	6	Transposase for insertion sequence element IS1081
	Rv1047	5	Probable transposase
	Rv1361c	3	PPE family protein PPE19
	Rv3892c	3	PPE family protein PPE69
*Mycobacterium bovis subsp*. bovis AF2122/97	Mb1076	6	IS1081 transposase
	Mb2540c	6	IS1081 transposase
	Mb3049c	6	Transposase
	Mb3142	6	Transposase
	Mb0097c	3	Hypothetical protein
	Mb0098c	3	Hypothetical protein
	Mb1006c	3	PE-PGRS family protein
	Mb1228	3	PPE family protein
	Mb1396c	3	PPE family protein

## Conclusion

Bacterial pathogens in clinical samples are difficult to detect because of their low concentration. Although more sensitive assays are being developed for detection of these pathogens, they are still limited by the low number of bacteria. In this study, we designed a new strategy that targets high copy number genes to improve sensitivity. The improved sensitivity is dependent on the copy number of a signature gene in bacteria. Since the genomes of nearly all known bacterial pathogens have been sequenced and annotated, it is possible to screen high copy target genes as signature sequences. Therefore, this strategy is universal and could be extended to other bacteria.

## Data Availability Statement

The original contributions presented in the study are included in the article/Supplementary Material, further inquiries can be directed to the corresponding author/s.

## Ethics Statement

The studies involving human participants were reviewed and approved by Ethics Committee of Insititute of Diaesae Control and Prevention, Academy of Military Medical Sciences. The patients/participants provided their written informed consent to participate in this study.

## Author Contributions

QD performed the main experiments. QW and JC analyzed the data and drafts the manuscript. HZ, ZC, and YW conceived the strategy and designed the experiment. JL, GS, and BL contributed to experiments and data analysis. All authors contributed to the article and approved the submitted version.

## Funding

This work was supported by the State Key Program of National Natural Science of China (U1808202), China Postdoctoral Science Foundation (2021M692233), NSFC International (regional) Cooperation and Exchange Program (31961143024), the National Key Program for Infectious Disease of China (2018ZX10101002-002), and Key Program of Inner Mongolia (2019ZD006).

## Conflict of Interest

The authors declare that the research was conducted in the absence of any commercial or financial relationships that could be construed as a potential conflict of interest.

## Publisher's Note

All claims expressed in this article are solely those of the authors and do not necessarily represent those of their affiliated organizations, or those of the publisher, the editors and the reviewers. Any product that may be evaluated in this article, or claim that may be made by its manufacturer, is not guaranteed or endorsed by the publisher.

## Supplementary Material

Supplementary Table 1 Primers sequences for these genes were designed and synthesized.

Supplementary Table 2 Genes with two or more alignment sites in the genomes.

Supplementary Table 3 Copy distribution of BMEI1001 among different species.

Supplementary Table 4 Copy distribution of BMEI0775 among different species.

Supplementary Table 5 Copy distribution of BMEI0027 among different species.

Supplementary Table 6 The 20 loci are distributed differently among these Brucella strains.
